# Urine *β*-2-Microglobulin, Osteopontin, and Trefoil Factor 3 May Early Predict Acute Kidney Injury and Outcome after Cardiac Arrest

**DOI:** 10.1155/2019/4384796

**Published:** 2019-05-07

**Authors:** Sigrid Beitland, Espen Rostrup Nakstad, Jens Petter Berg, Anne-Marie Siebke Trøseid, Berit Sletbakk Brusletto, Cathrine Brunborg, Christofer Lundqvist, Kjetil Sunde

**Affiliations:** ^1^Institute of Clinical Medicine, University of Oslo, P.O.Box 1072 Blindern, 0316 Oslo, Norway; ^2^Department of Anaesthesiology, Oslo University Hospital, P.O.Box 4950 Nydalen, 0424 Oslo, Norway; ^3^Norwegian National Unit for CBRNE Medicine, Oslo University Hospital, P.O.Box 4956 Nydalen, 0424 Oslo, Norway; ^4^Department of Medical Biochemistry, Oslo University Hospital, P.O.Box 4950 Nydalen, 0424 Oslo, Norway; ^5^Oslo Centre for Biostatistics and Epidemiology, Oslo University Hospital, P.O.Box 1122 Blindern, 0317 Oslo, Norway; ^6^Health Services Research Unit and Department of Neurology, Akershus University Hospital, P.O.Box 1000, 1478 Lørenskog, Norway

## Abstract

**Purpose:**

Acute kidney injury (AKI) is a common complication after out-of-hospital cardiac arrest (OHCA), leading to increased mortality and challenging prognostication. Our aim was to examine if urine biomarkers could early predict postarrest AKI and patient outcome.

**Methods:**

A prospective observational study of resuscitated, comatose OHCA patients admitted to Oslo University Hospital in Norway. Urine samples were collected at admission and day three postarrest and analysed for *β*-2-microglobulin (*β*2M), osteopontin, and trefoil factor 3 (TFF3). Outcome variables were AKI within three days according to the Kidney Disease Improving Global Outcome criteria, in addition to six-month mortality and poor neurological outcome (PNO) (cerebral performance category 3–5).

**Results:**

Among 195 included patients (85% males, mean age 60 years), 88 (45%) developed AKI, 88 (45%) died, and 96 (49%) had PNO. In univariate analyses, increased urine *β*2M, osteopontin, and TFF3 levels sampled at admission and day three were independent risk factors for AKI, mortality, and PNO. Exceptions were that *β*2M measured at day three did not predict any of the outcomes, and TFF3 at admission did not predict AKI. In multivariate analyses, combining clinical parameters and biomarker levels, the area under the receiver operating characteristics curves (95% CI) were 0.729 (0.658–0.800), 0.797 (0.733–0.861), and 0.812 (CI 0.750–0.874) for AKI, mortality, and PNO, respectively.

**Conclusions:**

Urine levels of *β*2M, osteopontin, and TFF3 at admission and day three were associated with increased risk for AKI, mortality, and PNO in comatose OHCA patients. This trail is registered with NCT01239420.

## 1. Introduction

Acute kidney injury (AKI) affects around 37% of initial cardiac arrest (CA) survivors; the condition is associated with increased morbidity and mortality [1, 2]. Uniform definitions of AKI based on serum creatinine and urine output are useful in clinical practice and research, for instance the Kidney Disease Improving Global Outcomes (KDIGO) criteria [3]. However, a major shortcoming of these definitions is that AKI is detected quite late, and early biomarkers that could predict AKI and even patient outcome would be useful [4].

There are several AKI biomarkers available in serum and/or urine, and many continue to be tested. Levels of *β*-2-microglobulin (*β*2M), osteopontin, and trefoil factor 3 (TFF3) are increased in urine due to AKI [5–7]. *β*2M is a protein widely distributed on the cell surface [5], osteopontin is a cytokine broadly expressed and upregulated during inflammation [6], whereas TFF3 is a small protein secreted by mucus-producing epithelial cells [7]. Previous studies indicate that *β*2M is a marker of glomerular and tubular injury, osteopontin is an inflammatory mediator, whereas TFF3 is upregulated in tubular injury [5–8].

The utility of AKI biomarkers has been tested in various cohorts of intensive care unit (ICU) populations, also after CA [9–12]. Former studies suggest that elevated levels of cystatin C and neutrophil gelatinase-associated lipocalin (NGAL) are associated with increased risk for AKI and adverse outcome postarrest [9–12], whereas the product of tissue inhibitor of metalloproteinase 2 (TIMP-2) and insulin-like growth factor binding protein 7 (IGFBP7) only seems to predict AKI [9].

Urine *β*2M, osteopontin, and TFF3 are rarely studied in ICU patients. Our aim was to examine whether these biomarkers could give an early prediction of AKI, mortality, and poor neurological outcome (PNO) after out-of-hospital cardiac arrest (OHCA). Our hypothesis was that increased urine *β*2M, osteopontin, and TFF3 levels were early markers of postarrest AKI and unfavourable patient outcome.

## 2. Materials and Methods

### 2.1. Study Design and Setting

This study is a post-hoc analysis of data from the prospective, observational Norwegian Cardiorespiratory Arrest Study (NORCAST) (NCT01239420), in which the primary aim was to assess early prediction of outcome after OHCA. Oslo University Hospital is a combined community and regional hospital in Norway with around 45,000 admissions per year. The study was approved by the Regional Committee for Medical Research Ethics of Eastern and Southern Norway (Approval number REK S-O A Ref 2010/1116a). Written, informed consent was obtained from the nearest family relative after admission, and later from all patients who regained consciousness and were competent to give consent within six months.

### 2.2. Study Population

Patients were consecutively enrolled according to predefined inclusion and exclusion criteria between September 8, 2010, and January 13, 2014. Inclusion criteria were adult (≥18 years) comatose OHCA patients with return of spontaneous circulation (ROSC). Exclusion criteria were known chronic kidney disease (CKD), patients who died within 24 hours of ICU stay, or for some reason did not receive active treatment. Patients were treated at Oslo University Hospital, Ullevål, according to a standard operating procedure (SOP) for OHCA patients. All patients were treated with targeted temperature management with a temperature set at 33° Celsius for 24 hours. Patients also received adjusted fluid therapy, vasopressor, and inotropic agents aiming for specified hemodynamic goals.

### 2.3. Study Definitions

OHCA was defined as absence of spontaneous respiration in a comatose patient receiving cardiopulmonary resuscitation. ROSC was identified as sustained electrical activity on the electrocardiogram generating a palpable pulse lasting longer than 20 minutes. AKI and chronic kidney disease (CKD) were classified according to the KDIGO guidelines [3, 13], but only data from the first three days of ICU stay were assessed, using both serum creatinine and urine output criteria. PNO was defined as a cerebral performance category (CPC) 3–5 [14]. Simplified Acute Physiology Score (SAPS) II was used to assess the severity of illness [15] and Sequential Organ Failure Assessment (SOFA) score to consider the extent of organ failures [16].

### 2.4. Data Collection

Clinical parameters were collected including patient demographics, prior health history, cause of arrest, and traditional prehospital CA data according to the Utstein template [17]. In-hospital information was obtained including routinely collected clinical and laboratory data. Additional urine samples were collected for the purpose of the biomarker analyses. Outcome data were obtained during an extensive neurological consultation six months postarrest which included CPC classification, originally described as the Glasgow outcome scale [14]. The scale was further dichotomised into good (CPC 1-2) and poor (CPC 3–5) neurological outcomes.

### 2.5. Biochemical Sampling and Analyses

Urine samples were collected from urine catheters at admission (0 to 6 hours postarrest) and day three. Samples were stored and analysed as previously described using the Bio-Plex Pro RBM Human Kidney Toxicity Assays panel 2 on the Bio-Plex 200 system (Bio-Rad Laboratories, Hercules, CA, USA) [9].

### 2.6. Statistical Methods

Univariate analyses were performed using Pearson's chi square test and Fisher's exact test. The association between potential risk factors and the outcomes (AKI, mortality and PNO) was quantified by odds ratio (OR) with 95% confidence interval (CI). In order to compare dichotomous and continuous risk factors, we dichotomized continuous variables using median values as cutoff levels to achieve equal number of observations in each group. Variables with *p* < 0.25 in the univariate analyses were assessed as candidates for the multivariate model. Independent risk factors were found using a multivariate logistic regression model with manual backward stepwise elimination procedure. We further estimated the correlation between risk factors and evaluated the predictive accuracy of the models assessing calibration and discrimination. The Hosmer and Lemeshow goodness-of-fit test was used to evaluate calibration. A statistically nonsignificant Hosmer and Lemeshow result (*p* > 0.05) suggests that the model predicts accurately on average. The area under receiver operating characteristics (AuROC) curve was used to evaluate discrimination. Acceptable discriminatory capability was defined as an AuROC above 0.7. In the calculation of hourly urine output, we lacked information on patient weight in some patients. Patients without recorded body weight were assumed to be 70 kg if female and 80 kg if male. There were some additional missing data, and these were handled using only available data. A power analysis was not performed since the measured AKI biomarkers were not the primary endpoint of the NORCAST study. Statistical analyses were performed using SPSS 21 for Windows (IBM SPSS, Chicago, IL, USA) and Stata 15 (Stata-Corp, College Station, TX, USA). Two-sided *p*-values less than 0.05 were considered statistically significant.

## 3. Results

### 3.1. Patient Characteristics and Event Rates

**Figure 1:**
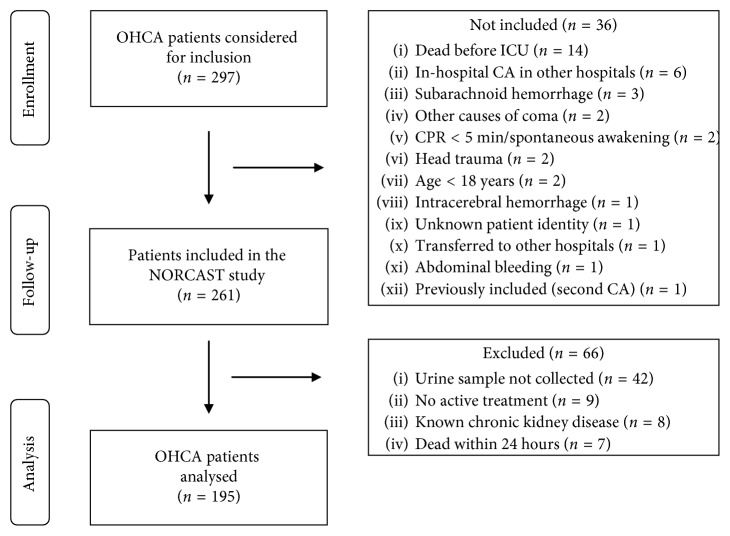
Flow chart of the study. OHCA: out-of-hospital cardiac arrest; NORCAST: Norwegian cardiorespiratory arrest study; ICU: intensive care unit; CA: cardiac arrest; CPR: cardiopulmonary resuscitation.

Of 297 OHCA patients eligible during the study period, 261 patients were included in the NORCAST study. Additional patients were excluded from this substudy because urine samples were not collected (*n*=42), they did not receive active treatment (*n*=9), had known CKD (*n*=8), or died within 24 hours of ICU stay (*n*=7) (Figure 1). There was no loss to follow-up.

In the cohort of 195 included patients, 165 (85%) were males and mean age was 60 (±14) years. CA was witnessed in 165 (85%) patients, 128 (66%) patients had initial shockable rhythm, and median time to ROSC was 25 (16–33) minutes. SOFA score on admission day was 10 (9–11), with organ-specific scores for central nervous system 4 (4-4), cardiovascular system 3 (3-4), respiratory system 3 (2–4), liver 0 (0-0), kidney 0 (0-1), and coagulation 0 (0-0), respectively. AKI developed in 88 (45%) patients, and renal replacement therapy (RRT) was used in 8 (4%). Overall, six-month outcome revealed that 88 (45%) died and 96 (49%) had PNO. Time from OHCA to death was 10 (3–20) days. The cause of death was considered to be certain cardial in 9 (10%) patients, likely cardial in 27 (31%) patients, certainly cerebral in 6 (7%) patients, likely cerebral in 34 (39%) patients, certainly not cardiocerebral in 10 (11%) patients, and unknown in 2 (2%) patients, respectively. Urine samples were collected from all patients at admission and 164 (84%) patients at day three.

### 3.2. Risk Factors for AKI

Many potential risk factors for AKI were found in univariate analysis (Table 1). Urine concentrations of *β*2M, osteopontin, and TTF3 were significantly higher in patients with AKI compared to those without AKI, except for *β*2M at day tree and TTF3 at admission (Table 1).

**Figure 2:**
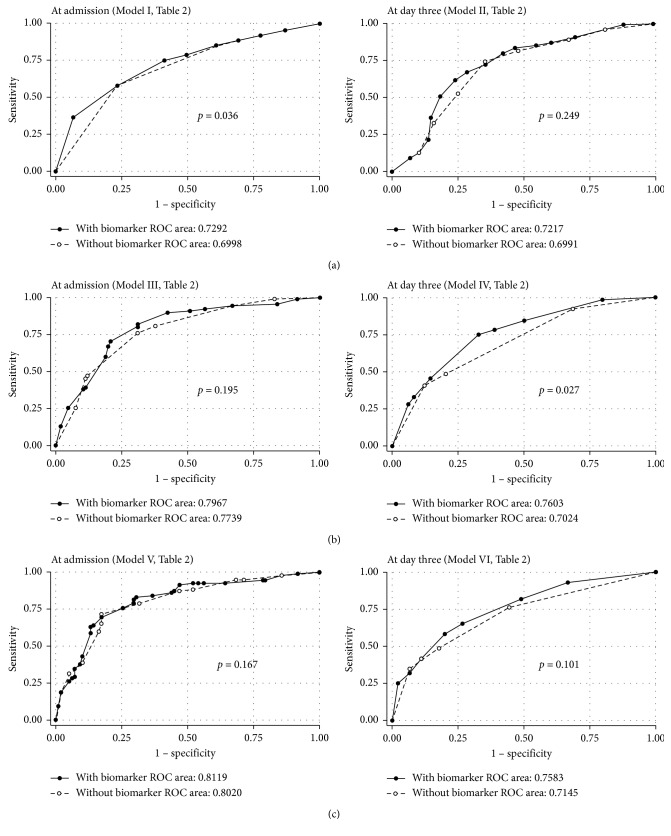
Area under the receiver operating characteristics curve (AuROC) plots for predictive models of acute kidney injury, mortality, and poor neurological outcome in out-of-hospital cardiac arrest patients. Presented *p* values are for comparison of models consisting of clinical parameters with and without acute kidney injury biomarkers measured in urine. (a) Predictors of acute kidney injury. (b) Predictors of mortality. (c) Predictors of poor neurological outcome.

In multivariate analyses, SOFA score ≥ 10 (OR 3.59, 95% CI 1.72–7.47), serum urea concentrations ≥ 6.7 mmol/L (OR 2.71, 95% CI 1.47–5.04), and urine *β*2M levels ≥ 2769 ng/mL (OR 2.14, 95% CI 1.16–3.96) were associated with the increased risk for AKI. In the best predictive model, AuROC was 0.729 (95% CI 0.658–0.800), indicating a good discriminating ability between patients with and without AKI (Table 2, Figure 2).

### 3.3. Risk Factors for Mortality and Poor Neurological Outcome

Univariate analyses revealed many potential risk factors for mortality (Table 3) and PNO (Table 4). Urine biomarker concentrations were higher in nonsurvivors compared to survivors and in patients with PNO compared with patients who had favourable neurological outcome. An exception was that *β*2M levels at day three did not predict mortality or PNO.

Factors associated with increased risk for mortality in multivariate analyses were serum urea concentrations ≥ 10 mmol/L (OR 2.16, 95% CI 1.11–4.20), absence of initial ventricular fibrillation/ventricular tachycardia (VT/VF) (OR 5.29, 95% CI 2.58–10.87), SOFA score ≥ 10 (OR 4.46, 95% CI 2.00–9.96), and urine *β*2M levels ≥ 2769 ng/mL (OR 2.65, 95% CI 1.36–5.18). In the best predictive model for mortality, AuROC was 0.797 (95% CI 0.733–0.861), indicating a good discriminating ability between survivors and nonsurvivors (Table 2, Figure 2).

In multivariate analyses, serum urea concentrations ≥ 6.7 mmol/L (OR 2.19, 95% CI 1.11–4.32), absence of initial VT/VF (OR 5.62, 95% CI 2.64–11.97), whole blood base excess (BE) levels < −7 mmol/L (OR 2.40, 95% CI 1.18–4.85), SOFA score ≥ 10 (OR 2.98, 95% CI 1.34–6.66), and urine *β*2M levels ≥ 2769 ng/mL (OR 2.10, 95% CI 1.04–4.21) were associated with increased risk for PNO. In the best predictive model for PNO, AuROC was 0.812 (95% CI 0.750–0.874), indicating a good discriminating ability between patients with good and poor neurological outcomes (Table 2 and Figure 2).

### 3.4. Additional Findings

Addition of biomarker levels to clinical parameters significantly increased the discriminating power in two out of six predictive models (Table 2 and Figure 2). All the predictive models presented had a not significant Hosmer and Lemeshow goodness-of-fit test, indicating satisfactory fit of the models (data not presented). Parameters excluded from all multivariate analyses were time to ROSC (due to 19 % missing data) and additionally whole blood bicarbonate and lactate levels which were strongly correlated (*r* > 0.7) to BE concentrations.

## 4. Discussion

In this prospective observational study of 195 OHCA patients, 45% developed AKI within three days and 51% had good outcome at six months. Increased urine concentrations of *β*2M, osteopontin, and TFF3 sampled at admission and day three were associated with increased risk for AKI, mortality, and PNO. Exceptions were that *β*2M measured at day three did not predict any of the assessed outcomes, and TFF3 at admission did not predict AKI. The ability to predict AKI, mortality, and PNO was good in models combining clinical parameters and biomarker concentrations, but the discriminating power was not uniformly improved by addition of biomarkers.

Many serum and urine biomarkers are able to predict AKI in ICU patients [18–25]. We, and others, have revealed that increased levels of cystatin C, NGAL, and (TIMP-2) × (IGFBP7) are risk factors for postarrest AKI [9–12]. In agreement with this, we found increased urine *β*2M, osteopontin, and TFF3 levels in patients with AKI compared to those without. Our observation that *β*2M measured at day three and TFF3 at admission were not associated with AKI might be due to the different time profile of these makers. Increased levels of urine *β*2M were associated with AKI in one small study of ICU patients [22], whereas osteopontin and TFF3 levels have not previously been studied in this patient group.

Increased concentrations of AKI biomarkers are associated with adverse outcomes after CA, related to reduced survival [9–12] and more frequent PNO [9, 12]. In comparison, we observed that elevated urine *β*2M, osteopontin, and TFF3 concentrations were associated with increased mortality and PNO, with the exception of *β*2M measured at day three. Our outcomes mortality and PNO were similar since most patients classified as PNO were dead. To our knowledge, no prior study has evaluated these biomarkers ability to predict outcome after OHCA. Urine *β*2M, osteopontin, and TFF3 have rarely been tested in humans, and their time profile of excretion is not fully clarified. Based on our study, one might speculate that *β*2M performs best when measured early (at admission), whereas TFF3 has the best discriminating power when measured later (at day three).

Unfortunately, AKI biomarkers seem to have limited practical value since biomarker levels overlap between patients with and without AKI and patients with good and poor outcomes [26–28]. In general, the diagnostic and prognostic utility of these biomarkers could be improved if the timing of sampling was optimized, but the ideal time point for urine *β*2M, osteopontin, and TFF3 is not known. Biomarker performance is also enhanced if used in a population with high probability of disease as we did in our patients. Another option is the use of predictive models with combined biomarker levels and clinical data [29, 30] although this did not uniformly improve the utility of biomarkers in our study.

This study has several limitations as it is difficult to ensure that patients do not have prearrest CKD. It is also challenging to time biomarker sampling to changes in kidney function. We used modified KDIGO criteria because they were applied for only three days with missing data of body weight in 29 patients. Measured biomarker concentrations at admission may be affected by urine present in the bladder prior to the arrest. Moreover, we were unable to compare the predictive ability of biomarkers at admission and day three since we lacked urine from day three in 31 patients. We were also unable to include time to ROSC in the multivariate analyses because data were missing in 37 patients. Furthermore, *β*2M may be instable in urine if the pH is below six, and this was not measured in the present study. Additionally, we have not controlled for presence of AKI when assessing the biomarkers ability to predict mortality and PNO. Finally, the study has limited sample size and probably restricted external validity. Strengths of the study are that the compared patients came from the same cohort and time period, with clear definitions of risk factors and outcome variables and no loss to follow-up.

## 5. Conclusions

This study of resuscitated comatose OHCA patients revealed that elevated urine *β*2M, osteopontin, and TFF3 levels at admission and day three were associated with increased risk for AKI, mortality, and PNO. Exceptions were that urine *β*2M concentrations measured at day three did not predict any of the assessed outcomes, and TFF3 at admission did not predict AKI. The biomarker changes observed in this study were probably markers of the multiple organ impact of cardiac arrest, but these biomarkers should not be used alone to direct patient care.

## Figures and Tables

**Table 1:** Univariate analyses of risk factors for acute kidney injury in resuscitated, comatose out-of-hospital cardiac arrest patients.

	Without AKI (*n*=107)	With AKI (*n*=88)	Risk factor for AKI	Crude OR (95% CI) for AKI	*p* value
*Baseline data*					
Age (years)	60.0 ± 13.7	60.2 ± 13.4	Age ≥ 60 years	0.90 (0.51 to 1.58)	0.710
Weight (kg)^a^ (*n*=166)	80.0 (75.0 to 90.0)	85.0 (80.0 to 94.5)	Weight ≥ 85 kg	1.73 (0.93 to 3.20)	0.083
Male (sex)	92 (86.0)	73 (83.0)	Female sex	1.26 (0.58 to 2.75)	0.560
Witnessed CA^a^ (*n*=194)	98 (92.5)	71 (80.7)	Unwitnessed CA	2.93 (1.20 to 7.17)	0.015
Bystander CPR	96 (89.7)	75 (85.2)	Not bystander CPR	1.51 (0.64 to 3.57)	0.342
ROSC time (min)^a^ (*n*=158)	22.0 (15.0 to 29.0)	30.0 (20.0 to 42.5)	Time to ROSC ≥ 25 min	2.16 (1.13 to 4.11)	0.018
Initial VF/VT^a^ (*n*=193)	76 (71.0)	52 (59.1)	Not initial VF/VT	1.71 (0.93 to 3.11)	0.081
SAPS II (score)	68.2 ± 10.1	73.1 ± 10.3	SAPS II score ≥ 69	1.92 (1.08 to 3.42)	0.026

*Admission day*					
Diuresis (L/day)	2.26 (1.82 to 3.28)	1.81 (1.43 to 2.45)	Diuresis < 1.93 L/day	3.69 (2.04 to 6.70)	<0.001
Fluid balance (L/day)	4.01 (2.79 to 5.77)	4.74 (3.50 to 6.30)	Fluid balance ≥ 4.45 L/day	1.49 (0.84 to 2.62)	0.169
S-Creatinine (*μ*·mol/L)	94.0 (81.3 to 105.0)	107.0 (94.0 to 140.0)	S-Creatinine ≥ 101 *μ*·mol/L	5.18 (2.80 to 9.59)	<0.001
S-Urea (mmol/L)	6.3 (5.2 to 7.5)	7.3 (5.8 to 9.6)	S-Urea ≥ 6.7 mmol/L	2.74 (1.53 to 4.91)	0.001
B-HCO_3_^−^ (mmol/L)	20.7 (18.3 to 22.8)	18.9 (16.4 to 21.2)	B-HCO_3_^−^ < 19.0 mmol/L	1.95 (1.08 to 3.52)	0.025
B-BE (mmol/L)	−5.6 (−9.1 to −3.6)	−8.9 (−12.4 to −6.1)	B-BE < −7.0 mmol/L	2.68 (1.50 to 4.80)	0.001
B-Lactate (mmol/L)	3.0 (1.7 to 6.5)	5.2 (2.9 to 9.3)	B-Lactate ≥ 4.1 mmol/L	1.92 (1.08 to 3.39)	0.025
SOFA (score)	10.0 (9.0 to 11.0)	11.0 (10.0 to 12.0)	SOFA score ≥ 10	3.73 (1.84 to 7.55)	<0.001

*Urine biomarkers* (*n*=195*at admission andn*=164*at day three*)					
Adm. *β*2M (ng/mL)	1856 (364 to 5356)	4393 (833 to 7986)	Adm. *β*2M ≥ 2769 ng/mL	2.09 (1.18 to 3.70)	0.012
Day 3 *β*2M (ng/mL)^a^	698 (109 to 3832)	412 (67 to 5428)	Day 3 *β*2M ≥ 627 ng/mL	0.77 (0.41 to 1.44)	0.408
Adm. osteopontin (ng/mL)	1876 (1382 to 2570)	2310 (1791 to 3104)	Adm. osteopontin ≥2068 ng/mL	2.36 (1.33 to 4.21)	0.003
Day 3 osteopontin (ng/mL)^a^	1403 (691 to 2395)	2539 (1254 to 4397)	Day 3 osteopontin ≥1683 ng/mL	2.17 (1.15 to 4.12)	0.017
Adm. TFF3 (ng/mL)	2160 (960 to 4084)	3229 (1653 to 5091)	Adm. TFF3, ng/mL ≥ 2694 ng/mL	1.68 (0.95 to 2.97)	0.073
Day 3 TFF3 (ng/mL)^a^	2448 (1130 to 4173)	3695 (2111 to 5853)	Day 3 TFF3, ng/mL ≥ 2910 ng/mL	2.32 (1.22 to 4.41)	0.010

*Outcome*					
Hospital RRT	0 (0.0)	8 (9.1)	Treatment with RRT		n.a.
Dead at 6 months	32 (29.9)	56 (63.6)	Death		n.a.
PNO at 6 months	37 (34.6)	59 (67.0)	Poor neurological outcomes		n.a.

Categorical data are presented as number (percent), continuous data with skewed distribution as median (interquartile range), and continuous data with normal distribution as mean (±standard deviation). Presented *p* values are from univariate Pearson's chi square analysis. AKI: acute kidney injury; OR: odds ratio; CI: confidence interval; *n*: number; CA: cardiac arrest; CPR: cardiopulmonary resuscitation; ROSC: return of spontaneous circulation; VF/VT: ventricular fibrillation/ventricular tachycardia; SAPS: simplified acute physiology score; S: serum; B: whole blood; HCO_3_^−^: bicarbonate; BE: base excess; SOFA: sequential organ failure assessment; Adm.: admission; *β*2M: *β*-2-microglobulin; TFF3: trefoil factor 3; RRT: renal replacement therapy; PNO: poor neurological outcome defined as cerebral performance category (CPC) 3–5; n.a.: not applicable. ^a^Data from some patients are missing.

**Table 2:** Multivariate analyses of risk factors for acute kidney injury, mortality, and poor neurological outcome in resuscitated, comatose out-of-hospital cardiac arrest patients.

	Covariates	Levels	Adjusted OR (95% CI)	*p* value^*∗*^	AuROC (95% CI) with biomarker	AuROC (95% CI) without biomarker	*p* value^*∗∗*^
*Risk factors for acute kidney injury*							
Model I (*n*=195)	SOFA score, day 0	≥/<10	3.59 (1.72 to 7.47)	0.001	0.729 (0.658 to 0.800)	0.700 (0.630 to 0.770)	0.036
	S-Urea, day 0	≥/<6.7 mmol/L	2.71 (1.47 to 5.04)	0.001			
	Admission U-*β*2M	≥/<2769 ng/mL	2.14 (1.16 to 3.96)	0.015			
Model II (*n*=142)	S-Urea, day 0	≥/<6.7 mmol/L	2.18 (1.05 to 4.53)	0.036	0.722 (0.636 to 0.807)	0.699 (0.612 to 0.786)	0.249
	Weight^a^	≥/<85 kg	2.09 (1.00 to 4.38)	0.050			
	B-BE	≥/<−7.0 mmol/L	2.49 (1.20 to 5.20)	0.015			
	U-Osteopontin, day 3	≥/<1683 ng/mL	2.07 (1.00 to 4.29)	0.050			

*Risk factors for mortality*							
Model III (*n*=193)	S-Urea, day 0	≥/<6.7 mmol/L	2.16 (1.11 to 4.20)	0.023	0.797 (0.733 to 0.861)	0.774 (0.709 to 0.839)	0.195
	Not initial VT/VF^a^	No/yes	5.29 (2.58 to 10.87)	<0.001			
	SOFA score, day 0	≥/< 10	4.46 (2.00 to 9.96)	<0.001			
	Admission U-*β*2M	≥/< 2769 ng/mL	2.65 (1.36 to 5.18)	0.004			
Model IV (*n*=162)	Not initial VT/VF^a^	No/yes	3.65 (1.71 to 7.76)	0.001	0.760 (0.687 to 0.833)	0.702 (0.627 to 0.778	0.027
	SOFA score, day 0	≥/<10	3.31 (1.43 to 7.69)	0.005			
	U-TTF3, day 3	≥/<2910 ng/mL	3.40 (1.66 to 6.95)	0.001			

*Risk factors for poor neurological outcomes*							
Model V (*n*=193)	S-Urea, day 0	≥/<6.7 mmol/L	2.19 (1.11 to 4.32)	0.024	0.812 (0.750 to 0.874)	0.802 (0.740 to 0.864)	0.167
	Not initial VT/VF^a^	No/yes	5.62 (2.64 to 11.97)	<0.001			
	B-BE	≥/<−7.0 mmol/L	2.40 (1.18 to 4.85)	0.015			
	SOFA score, day 0	≥/< 10	2.98 (1.34 to 6.66)	0.008			
	Admission U-*β*2M	≥/< 2769 ng/mL	2.10 (1.04 to 4.21)	0.038			
Model VI (*n*=162)	Not initial VT/VF^a^	No/yes	3.83 (1.78 to 8.24)	0.001	0.758 (0.685 to 0.832)	0.715 (0.638 to 0.791)	0.101
	B-BE	≥/<−7.0 mmol/L	2.95 (1.46 to 5.97)	0.003			
	U-TTF3, day 3	≥/<2910 ng/mL	3.15 (1.56 to 6.40)	0.001			

Data are from multivariate logistic regression analysis. OR: odds ratio; CI: confidence interval; AuROC: area under the curve in the receiver operating characteristics curve; *n*: number; SOFA: sequential organ failure assessment; *β*2M: *β*-2-microglobulin; S: serum; U: urine; B: whole blood; BE: base excess; VF/VT: ventricular fibrillation/ventricular tachycardia; TFF3: trefoil factor 3. ^*∗*^*p* values for the adjusted odds ratio. ^*∗∗*^*p* values from comparing the AuROC with and without biomarkers. ^a^Data from some patients are missing.

**Table 3:** Univariate analyses of risk factors for mortality in resuscitated, comatose out-of-hospital cardiac arrest patients.

	Survivors (*n*=107)	Nonsurvivors (*n*=88)	Risk factor for mortality	Crude OR (95% CI) for mortality	*p* value
*Baseline data*					
Age (years)	59.1 ± 13.1	61.4 ± 14.1	Age ≥ 60 years	1.37 (0.77 to 2.41)	0.283
Weight (kg)^a^ (*n*=166)	83.0 (75.0 to 93.0)	85.0 (75.0 to 90.0)	Weight ≥ 85 kg	1.35 (0.73 to 2.51)	0.335
Male (sex)	94 (87.9)	71 (80.7)	Female sex	1.73 (0.79 to 3.80)	0.167
Witnessed CA^a^ (*n*=194)	101 (95.3)	68 (77.3)	Unwitnessed CA	5.94 (2.13 to 16.59)	<0.001
Bystander CPR	94 (87.9)	77 (87.5)	Not bystander CPR	1.03 (0.44 to 2.44)	0.941
ROSC time (min)^a^ (*n*=158)	19.0 (12.0 to 29.0)	30.0 (23.0 to 44.0)	Time to ROSC ≥ 25 min	3.28 (1.68 to 6.40)	<0.001
Initial VF/VT^a^ (*n*=193)	86 (81.1)	42 (48.3)	Not initial VF/VT	4.61 (2.42 to 8.76)	<0.001
SAPS II (score)	68.3 ± 10.4	73.0 ± 10.0	SAPS II score ≥ 69	1.62 (0.91 to 2.87)	0.099

*Admission day*					
Diuresis (L/day)	2.03 (1.75 to 2.84)	1.81 (1.43 to 2.45)	Diuresis < 1.93 L/day	1.99 (1.12 to 3.53)	0.018
Fluid balance (L/day)	4.05 (2.37 to 5.74)	4.75 (3.50 to 6.39)	Fluid balance ≥ 4.45 L/day	1.92 (1.08 to 3.39)	0.025
S-Creatinine (*μ*·mol/L)	98.0 (84.0 to 114.0)	107.5 (94.3 to 140.0)	S-Creatinine ≥ 101 *μ*·mol/L	1.76 (1.00 to 3.11)	0.051
S-Urea (mmol/L)	6.3 (5.1 to 7.7)	7.3 (5.8 to 9.7)	S-Urea ≥ 6.7 mmol/L	1.93 (1.09 to 3.43)	0.023
B-HCO_3_^−^ (mmol/L)	20.6 (18.9 to 22.4)	19.0 (16.6 to 21.2)	B-HCO_3_^−^ < 19.0 mmol/L	2.82 (1.55 to 5.14)	0.001
B-BE (mmol/L)	−5.7 (−8.4 to −3.7)	−8.8 (−12.4 to −6.0)	B-BE < −7.0 mmol/L	3.51 (1.94 to 6.35)	<0.001
B-Lactate (mmol/L)	3.3 (1.7 to 5.9)	5.2 (2.9 to 9.3)	B-Lactate ≥ 4.1 mmol/L	2.48 (1.39 to 4.43)	0.002
SOFA (score)	10.0 (8.0 to 11.0)	11.0 (10.0 to 12.0)	SOFA score ≥ 10	4.26 (2.07 to 8.75)	<0.001

*Urine biomarkers* (*n*=195*at admission andn*=164*at day three*)					
Adm. *β*2M (ng/mL)	1840 (361 to 5356)	4840 (1117 to 9415)	Adm. *β*2M ≥ 2769 ng/mL	2.27 (1.28 to 4.05)	0.005
Day 3 *β*2M (ng/mL)^a^	628 (90 to 4678)	617 (67 to 4884)	Day 3 *β*2M ≥ 627 ng/mL	1.04 (0.56 to 1.95)	0.898
Adm. osteopontin (ng/mL)	1876 (1334 to 2588)	2553 (1814 to 3251)	Adm. osteopontin ≥ 2068 ng/mL	1.99 (1.12 to 3.53)	0.018
Day 3 osteopontin (ng/mL)^a^	1345 (590 to 2539)	2386 (1317 to 4397)	Day 3 osteopontin ≥ 1683 ng/mL	2.69 (1.41 to 5.15)	0.002
Adm. TFF3 (ng/mL)	2010 (1066 to 3964)	3400 (1827 to 5110)	Adm. TFF3, ng/mL ≥ 2694 ng/mL	2.17 (1.22 to 3.85)	0.008
Day 3 TFF3 (ng/mL)^a^	2447 (1045 to 987)	3784 (2384 to 5969)	Day 3 TFF3, ng/mL ≥ 2910 ng/mL	3.61 (1.86 to 7.02)	<0.001

*Outcome*					
Hospital RRT	3 (2.8)	5 (5.7)	Treatment with RRT	2.09 (0.48 to 9.01)	0.314
AKI within 3 days	32 (29.9)	56 (63.6)	Presence of AKI	4.10 (2.52 to 7.46)	<0.001
PNO at 6 months	8 (7.5)	88 (100.0)	Poor neurological outcome		n.a.

Categorical data are presented as number (percent), continuous data with skewed distribution as median (interquartile range), and continuous data with normal distribution as mean (±standard deviation). Presented *p* values are from univariate Pearson's chi square analysis. OR: odds ratio; CI: confidence interval; *n*: number; CA: cardiac arrest; CPR: cardiopulmonary resuscitation; ROSC: return of spontaneous circulation; VF/VT: ventricular fibrillation/ventricular tachycardia; SAPS: simplified acute physiology score; S: serum; B: whole blood; HCO_3_^−^: bicarbonate; BE: base excess; SOFA: sequential organ failure assessment; Adm.: admission; *β*2M: *β*-2-microglobulin; TFF3: trefoil factor 3; RRT: renal replacement therapy; AKI: acute kidney injury; PNO: poor neurological outcome defined as cerebral performance category (CPC) 3–5; n.a.: not applicable. ^a^Data from some patients are missing.

**Table 4:** Univariate analyses of risk factors for poor neurological outcome in resuscitated, comatose out-of-hospital cardiac arrest patients.

	Good neurological outcome (*n*=99)	PNO (*n*=96)	Risk factor for PNO	Crude OR (95% CI) for PNO	*p* value
*Baseline data*					
Age, years	59.2 ± 16.4	61.0 ± 14.7	Age ≥ 60 years	1.32 (0.75 to 2.32)	0.339
Weight (kg)^a^ (*n*=166)	83.0 (75.0 to 93.3)	85.0 (75.0 to 90.0)	Weight ≥ 85 kg	1.28 (0.69 to 2.36)	0.428
Male (sex)	87 (87.9)	78 (81.3)	Female sex	1.67 (0.76 to 3.69)	0.200
Witnessed CA^a^ (*n*=194)	94 (95.9)	75 (78.1)	Unwitnessed CA	6.58 (2.17 to 20.00)	<0.001
Bystander CPR	86 (86.9)	85 (88.5)	Not bystander CPR	0.86 (0.36 to 2.02)	0.722
ROSC time (min)^a^ (*n*=158)	22.5 (12.0 to 29.0)	30.0 (23.0 to 40.0)	Time to ROSC ≥ 25 min	3.16 (1.63 to 6.10)	0.001
Initial VF/VT^a^ (*n*=193)	82 (83.7)	46 (48.4)	Not initial VF/VT	5.46 (2.79 to 10.67)	<0.001
SAPS II (score)	67.8 ± 10.4	73.1 ± 9.9	SAPS II score ≥ 69	2.01 (1.13 to 3.56)	0.017

*Admission day*					
Diuresis (L/day)	2.03 (1.77 to 2.86)	1.81 (1.43 to 2.50)	Diuresis < 1.93 L/day	1.98 (1.12 to 3.50)	0.018
Fluid balance (L/day)	3.97 (2.58 to 5.64)	4.80 (3.46 to 6.45)	Fluid balance ≥ 4.45 L/day	2.07 (1.17 to 3.66)	0.012
S-Creatinine (*μ*·mol/L)	96.0 (84.0 to 113.0)	107.5 (94.0 to 139.3)	S-Creatinine ≥ 101 *μ*·mol/L	1.90 (1.08 to 3.36)	0.026
S-Urea (mmol/L)	6.3 (5.3 to 7.8)	7.1 (5.7 to 9.6)	S-Urea ≥ 6.7 mmol/L	1.90 (1.08 to 3.36)	0.026
B-HCO_3_^−^ (mmol/L)	20.6 (18.9 to 22.6)	19.0 (17.1 to 21.2)	B-HCO_3_^−^ < 19.0 mmol/L	2.58 (1.42 to 4.71)	0.002
B-BE (mmol/L)	−5-6 (−8.2 to −3.6)	−8.5 (−12.0 to −6.0)	B-BE < −7.0 mmol/L	4.18 (2.30 to 7.68)	<0.001
B-Lactate (mmol/L)	2.8 (1.6 to 5.4)	5.1 (3.1 to 9.1)	B-Lactate ≥ 4.1 mmol/L	2.68 (1.50 to 4.77)	0.001
SOFA (score)	10.0 (9.0 to 11.0)	11.0 (10.0 to 12.0)	SOFA score ≥ 10	3.66 (1.85 to 7.24)	<0.001

*Urine biomarkers (n*=195*at admission andn*=164*at day three)*					
Adm. *β*2M (ng/mL)	1458 (346 to 5356)	4172 (1117 to 8616)	Adm. *β*2M ≥ 2769 ng/mL	2.25 (1.27 to 3.99)	0.005
Day 3 *β*2M (ng/mL)^a^	630 (110 to 4945)	570 (66 to 4598)	Day 3 *β*2M ≥ 627 ng/mL	1.00 (0.54 to 1.84)	0.986
Adm. osteopontin, ng/mL	1871 (1266 to 2570)	2293 (1841 to 3151)	Adm. osteopontin ≥2068 ng/mL	2.35 (1.32 to 4.17)	0.003
Day 3 osteopontin (ng/mL)^a^	1344 (570 to 2648)	2239 (1270 to 4148)	Day 3 osteopontin ≥1683 ng/mL	2.60 (1.38 to 4.91)	0.003
Adm. TFF3 (ng/mL)	1982 (858 to 3743)	3400 1827 to 5110)	Adm. TFF3, ng/mL ≥ 2694 ng/mL	2.35 (1.32 to 4.17)	0.003
Day 3 TFF3 (ng/mL)^a^	2447 (961 to 3820)	3695 (2163 to 5622)	Day 3 TFF3, ng/mL ≥ 2910 ng/mL	3.07 (1.62 to 5.84)	0.001

*Outcome*					
Hospital RRT	2 (2.0)	6 (6.3)	Treatment with RRT	3.28 (0.64 to 16.39)	0.137
AKI within 3 days	29 (29.3)	59 (61.5)	Presence of AKI	3.85 (2.12 to 6.94)	<0.001
Dead at 6 months	0 (0.0)	88 (91.7)	Death		n.a.

Categorical data are presented as number (percent), continuous data with skewed distribution as median (interquartile range), and continuous data with normal distribution as mean (±standard deviation). Presented *p* values are from univariate Pearson's chi square analysis. PNO: poor neurological outcome defined as cerebral performance category (CPC) 3–5; OR: odds ratio; CI: confidence interval; *n*: number; CA: cardiac arrest; CPR: cardiopulmonary resuscitation; ROSC: return of spontaneous circulation; VF/VT: ventricular fibrillation/ventricular tachycardia; SAPS: simplified acute physiology score; S: serum; B: whole blood; HCO_3_^−^: bicarbonate; BE: base excess; SOFA: sequential organ failure assessment; Adm.: admission; *β*2M: *β*-2-microglobulin; TFF3: trefoil factor 3; RRT: renal replacement therapy; AKI: acute kidney injury; n.a.: not applicable. ^a^Data from some patients are missing.

## Data Availability

The data used to support the findings of this study are available from the corresponding author upon request with the approval of Oslo University Hospital.
